# Hydrogen Sulfide (H_2_S) Releasing Capacity of Isothiocyanates from *Moringa oleifera* Lam.

**DOI:** 10.3390/molecules23112809

**Published:** 2018-10-29

**Authors:** Xiangshe Wang, Yunjiao Liu, Xingdi Liu, Yi Lin, Xueqin Zheng, Yuyun Lu

**Affiliations:** 1 Institute of Tropical Agriculture and Forestry, Hainan University, No. 58, Renmin Avenue, Haikou 570228, Hainan, China; wangxiangshe@itbb.org.cn; 2 Institute of Tropical Bioscience and Biotechnology, Chinese Academy of Tropical Agricultural Sciences, No. 4, Xueyuan Road, Haikou 571101, Hainan, China; liuxingdi@itbb.org.cn; 3 Food Science and Technology Programme, Department of Chemistry, National University of Singapore, 3 Science Drive 3, Singapore 117543, Singapore; liuyunjiaoyj@gmail.com (Y.Liu.); linyi@u.nus.edu (Y.Lin.)

**Keywords:** *Moringa oleifera* Lam., glucosinolates, isothiocyanates, hydrogen sulfide, lead acetate

## Abstract

*Moringa oleifera* Lam. is rich in phytochemical compounds especially glucosinolates (GSs) and isothiocyanates (ITCs), which are active compounds for cancer chemoprevention benefits of Brassicaceae vegetables. In this study, we determined the total contents of GSs and ITCs and their specific profiles in different *Moringa* tissues including seeds, stems, leaves and roots. Seeds (seeds with shell and seed kernel) showed significantly higher levels of total GSs and ITCs than that of other *Moringa* tissues. The hydrogen sulfide (H_2_S) releasing capacity of total ITCs extracted from different *Moringa* tissues was determined by lead (II) acetate assay in 24-well plates. The H_2_S releasing capacity of different *Moringa* tissues were evaluated and compared. *Moringa* seeds showed the highest H_2_S releasing capacity, followed by roots, leaves and stems. Our results suggest that *Moringa* based foods may exhibit health benefits due to its GSs and ITCs contents that are the precursors for H_2_S, in addition to the recognized action mechanisms of ITCs.

## 1. Introduction

Hydrogen sulfide (H_2_S), with the characteristic foul odour of rotten egg, has been regarded as a new pleiotropic endogenous gasotransmitter besides nitric oxide (NO) and carbon monoxide (CO). Numerous studies showed that it might be beneficial to human health in neuroprotection, antioxidant, anticancer and blood vessel relaxation [1,2,3,4]. A previous study reported that H_2_S plays a key role in regulating cardiovascular homeostasis, acting as a direct relaxing regent in the vascular smooth muscle [5]. H_2_S has also been verified the positive effects in the nervous system in synaptic modulation via interacting with ion channels, second messengers and modifying sulfhydryl groups of proteins [6]. In mammals, H_2_S is mainly endogenously produced from L-cysteine under the catalysis of related enzymes including cysteine aminotransferase (CAT), 3-mercaptopyruvate sulphur transferase (3-MST), cystathionine *β*-synthase (SBC) and cystathionine *γ*-lyase (CSE) [7]. The benefits of endogenous H_2_S in human health highlight its great usefulness in the functional regulation. Therefore, exogenous H_2_S donors are viewed as useful tools for basic studies and promising drugs or active ingredients of functional foods.

**Figure 1:**
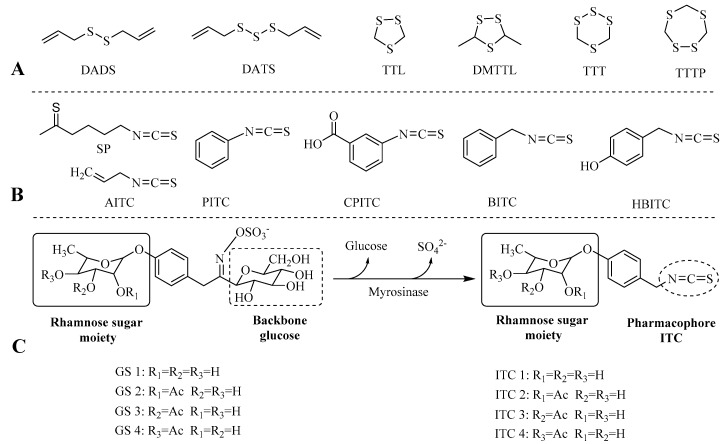
(**A**) Chemical structure of dietary polysulfur compounds; DADS, diallyl disulfide; DATS, diallyl trisulfide; TTL, 1,2,4-trithiolane; DMTTL, 3,5-dimethyl-1,2,4-trithiolane; TTT, 1,2,3,5-tetrathiane; TTTP, 1,2,3,6-tetrathiepane; (**B**) Chemical structures of isothiocyanates; SP, sulforaphane; AITC, allyl isothiocyanate; PITC, phenyl isothiocyanate; CPITC, 3-carboxyphenyl isothiocyanate; BITC, benzyl isothiocyanate; HBITC, 4-hydroxybenzyl isothiocyanate; (**C**) Enzymatic conversion of *Moringa* glucosinolates to isothiocyanates.

Various synthetic H_2_S donors have been developed for research and therapeutic purposes [8,9]. They can only be used to facilitate the understanding of H_2_S physiology and pharmacology, and are not suitable for drugs or active ingredient of functional foods. Recently, more and more naturally occurring H_2_S donors (polysulfides) from vegetable and fruits (e.g., garlic, stinky beans and durian) are widely investigated. These dietary polysulfides, including diallyl disulfide, diallyl trisulfide and cyclic polysulfides (Figure 1A), act as H_2_S donors with different releasing mechanisms, requiring the presence of reduced glutathione [10,11].

In addition, some other dietary organosulfur compounds, such as synthetic aryl isothiocyanates (e.g., phenyl isothiocyanate (PITC), carboxyphenyl isothiocyanate (CPITC), benzyl isothiocyanate (BITC) and 4-hydroxybenzyl isothiocyanate (HBITC)), as well as allyl isothiocyanate (AITC) (Figure 1B), have also been demonstrated to act as H_2_S donor agents [12,13]. Isothiocyanates (ITCs) are commonly known for their strong chemopreventive and anti-inflammatory effects due to their abilities in modulation of oxidative stress by induction of phase II detoxifying enzymes in liver [14,15,16]. Additionally, ITCs have been verified as the activators of the potassium channel, which plays a pivotal role in vascular smooth muscle (e.g., vasodilation) and pain modulation, clearly attributing to the release of H_2_S [17,18,19]. The bioactivity of ITCs is attributed to its pharmacophore (–N=C=S group, Figure 1C), which could cleave the disulfide bonds in proteins and react with amino acid such as cysteine [16,20]. The other part of the molecule also plays roles for stability, polarity, and volatility [16].

Naturally occurring ITCs are rich in the plant order Brassicales and formed from glucosinolates (GSs) under the action of myrosinase (*β*-thioglucosidase) [21,22,23]. Most ITCs are volatile viscous oils and unstable at room temperature, being spontaneously converted to inactive intermediates with relatively high degradation rates. In contrast to the volatile unstable ITCs from crucifers, ITCs from *Moringa oleifera*, from the corresponding *Moringa* GSs (Figure 1C), showed a solid form and high stable ability at room temperature, which was ascribed to the unique rhamnose moiety in the 4-phenyl moiety of the molecule [16,24].

*Moringa* species, especially *M. oleifera*, from the Moringaceae family, are traditional medical plants originating from India and Africa. They are often important famine foods and are now grown around the world because of their high tolerance to arid conditions due to their very large tuberous roots [22]. Almost all the tissues of *Moringa* species (e.g., roots, leaves, flowers, green pods and seeds) can be served as human foods and additional stems and petioles are used for animal feed. Seeds contain 30–40% oil that is high in oleic acid, which can be served as cooking oil. The defatting residues of seeds contain over 60% of protein, which can be easily extracted and used for cleaning contaminated water and slurry materials [22,25,26]. The leaves contain complete essential amino acids and are rich in proteins (ca. 27% by dry weight), vitamins and minerals [24,25]. The root and tree bark are a good source of tanning agents [27].

*Moringa* has been used medicinally throughout the centuries to treat a multitude of acute and chronic diseases [24]. Various phytochemical compounds (e.g., flavonols, chlorogenic acids, GSs and ITCs), obtained from different parts of the plant, have been verified to have an antimicrobial, anti-inflammatory, anticancer, antidiabetic, anti-hypertension and antispasmolytic effects [15,25,27,28]. Among these metabolites, GSs and ITCs are believed to be responsible for their health benefits. The aim of this study is to investigate the H_2_S releasing possibility and compare the H_2_S releasing capacity of ITCs extracted from different *Moringa* tissues (seeds, stems, leaves and roots).

## 2. Results and Discussion

Natural H_2_S donors from dietary sources may play a positive role for health promotion. While *Moringa* leaves have been promoted as food with many health promotion properties, the rich contents of GSs and ITCs have not been studied in depth. We analysed different *Moringa* tissues (seeds, stems, leaves and roots) for their total GSs and total ITCs contents, their GSs and ITCs profiles (HPLC and liquid chromatography-mass spectra (LC-MS) characterization) and H_2_S releasing capacity.

### 2.1. Extraction and Characterization of GSs and ITCs in Different Moringa Tissues

Currently, there were two existing GS extraction methods including desulfatation and intact GS extraction. However, the chromatography of GS extracts of *Moringa* leaves using the two extraction methods revealed completely different GS profiles [29]. Moreover, the precision of determination of GSs based on desulfatation method was significantly lower than that of intact GS extraction method. Furthermore, the desulfatation method resulted in the formation of artefact GSs and loss of the acetylated GSs [29]. All these indicated that the desulfatation method is not suitable for the analysis of GSs in *M. oleifera* samples. Therefore, the intact GSs extraction method was selected and used in this study.

**Figure 2:**
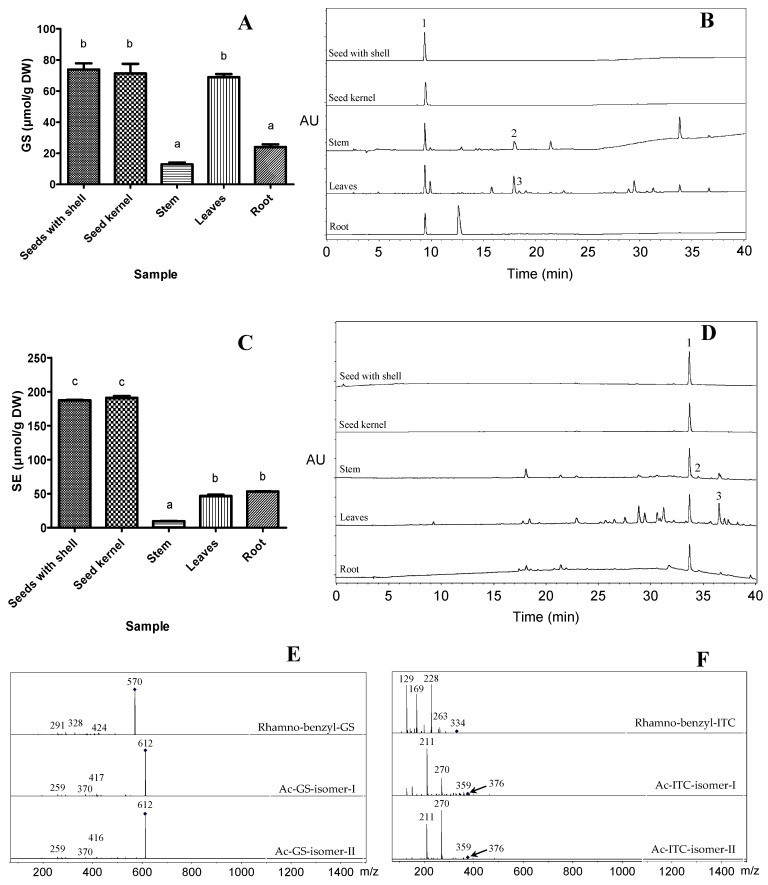
Total contents of glucosinolates (**A**) and HPLC chromatogram of extracted glucosinolates in different *Moringa* tissues at 227 nm (**B**); total contents of isothiocyanates (**C**) and HPLC chromatogram of extracted isothiocyanates in different *Moringa* tissues at 280 nm (**D**); MS^2^ spectra of GSs (**E**) and ITCs (**F**) in different *Moringa* tissues.

*Moringa* seeds (seeds with shell and seed kernels) and leaves showed significantly higher levels of total GSs than that of stem and root (Figure 2A). The HPLC chromatography and MS^2^ spectra of extracted GSs from different *Moringa* tissues is shown in Figure 2B,E, respectively. All *Moringa* tissues had essentially the same GSs profiles as previously reported [22,30]. 4-*O*-(α-l-rhamnopyranosyloxy)-benzylglucosinolate (Rhamno-benzyl-GS) was detected in all of the *Moringa* tissues (Figure 2B, Table 1). In seeds and roots, only rhamno-benzyl-GS was detected, one additional Ac-GS isomer was detected in stems and two additional Ac-GS isomers were detected in the leaves (Figure 2B). Our results were consistent with several previous studies, in that only rhamno-benzyl-GS was identified in seeds [22,31]. Unexpectedly, we did not find the third Ac-GS isomer in all *Moringa* tissues; this might be due to the conversion of the third Ac-GS isomer to the two detected because the acetyl group was able to move between the three hydroxyl groups of the rhamnopyranose [29]. Another study additionally reported a similar phenomenon for acetylated sialic acids, and that the re-arrangement of acetyl groups on sialic acids was affected by temperature and pH [32].

*Moringa* GSs contain an additional sugar moiety in the aglycone/ITC portion of the molecule. They can be converted in situ to the corresponding *Moringa* ITCs by the action of myrosinase (Figure 1C). This is consistent with the results of ITCs profiles, in that rhamno-benzyl ITC was detected in all *Moringa* tissues, and one additional Ac-ITC isomer was detected in the stem and two additional Ac-ITC isomers were detected in the leaves (Figure 2D,F, Table 2). In addition, *Moringa* seeds (seeds with shell and seed kernel, >180 μmol SE/g DW) showed significantly higher levels of total ITCs than that of the root, leaves and stem (Figure 2C).

GSs and the corresponding ITCs are receiving increasing interest for their numerous biological and pharmacological effects and useful applications in many aspects of human health [33,34]. A low micromolar concentration of ITCs could reduce the nitric oxide (NO) formation in macrophages, and therefore, contribute to its anti-inflammatory effect [35]. On the other hand, different ligand types of ITCs may show different activities. For example, the rhamno-benzyl-ITC showed more efficacy in inhibiting nuclear factor kappa B (NF-κB) and causing apoptosis than that of sulforaphane [36].

**Figure 3:**
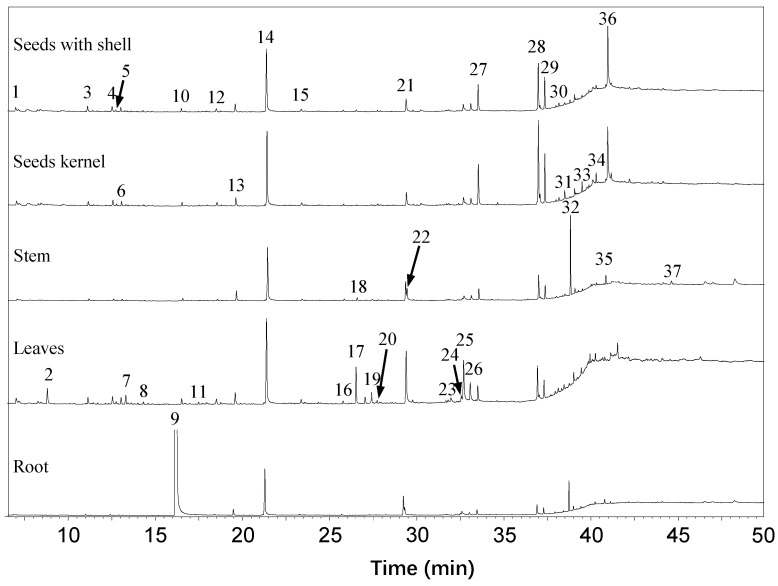
Volatile compounds identified by gas chromatography-mass spectrometry (GC-MS) in different *Moringa* tissues.

Some previous studies reported that there are other volatile ITCs in *Moringa* tissues [37,38,39], for example, 1-methylpropyl ITC, benzyl ITC, isopropyl ITC and isobutyl ITC were found in *Moringa* flowers (*Moringa oleifera*), leaves and seeds (*Moringa peregrine*). We also tried to find these volatile ITCs in different *Moringa* tissues. Unexpectedly, most of the detected volatile compounds were hydrocarbons, aldehydes and alcohols; we could only find m-tolyl ITC in *Moringa* root samples (Figure 3, Table 3). This might be due to difference of samples origin, growing conditions, as well as extraction methods used. In addition, these ITCs are commonly present, as volatile oils may not be stable at room temperatures [16,24,39].

### 2.2. H_2_S Releasing Capacity of ITC Extracts from Different Moringa Tissues

Lead acetate paper has been used to qualitative and quantitative analysis of the production of H_2_S due to the affinity of divalent lead and sulfide to form black lead sulfide (PbS) precipitate [11,40]. In addition, this method is easier to be conducted. Their method was followed with minor modifications.

**Figure 4:**
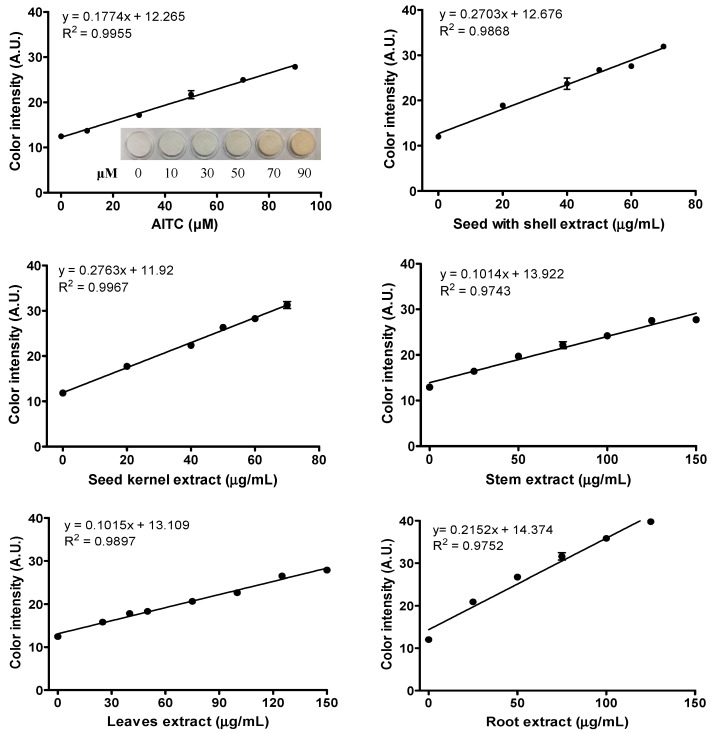
Dose response curves of H_2_S releasing capacity of extracted isothiocyanates from different *Moringa* tissues.

The linear dose response curves (R^2^ > 0.97) for different concentrations of AITC or ITC extracts from different *Moringa* tissues are shown in Figure 4. Here, we defined the AITC equivalent (AITC-E) as a unit to compare the H_2_S releasing capacity of ITC extracts from different *Moringa* tissues to that of AITC. The AITC-E value was used to compare the results among different batches and tissues. In the assay, the L-cysteine solution (10 mM) was prepared freshly in double strong phosphate buffer (PBS) and distributed into a 24-well plate. After that, different concentrations of AITC or ITC extracts from different *Moringa* tissues dissolved in dimethylformamide (DMF) was transferred into each well and mixed gently. The AITC-E value indicates the relative potency of H_2_S releasing capacity of ITC extracts compared to AITC. This assay showed consistent results in different test runs and is a reliable method for quantitative comparison of H_2_S releasing activity of dietary H_2_S donors. In addition, the advantage of lead acetate paper assay than other methods (e.g., GC-MS) is because it can detect H_2_S in the headspace of the solution, and therefore, it can avoid matrix interference [11].

To normalize the H_2_S releasing activity from different *Moringa* tissues, the AITC-E per 100 g freeze-dried *Moringa* tissues was calculated, taking the oil yields and total ITC yields into consideration (Table 4). Seed kernels showed the highest AITC-E value, followed by seeds with shell, roots, leaves and stems. The high AITC-E for seed kernels was largely due to its high yield of the ITC extract from the defatted seed kernel (Table 4).

## 3. Materials and Methods

### 3.1. Plant Material and Chemicals

Fresh leaves, stems, seeds and roots of *M. oleifera* were collected from the *Moringa* Planting Base, located at the Chengmai Jinma Avenue, Haikou, Hainan, China on 24 February 2018. All the materials were freeze-dried once collected from the tree. All the samples were blended (Blender Philips HR2095/3) into powder separately and stored in −20 °C. A voucher specimen (voucher No: lm2013042304) was deposited in the Institute of Tropical Bioscience and Biotechnology, Academy of Science in Haikou, China.

Methanol, ethyl acetate (EA), trifluoroacetic acid (TFA), acetic acid, dimethylformamide (DMF), sodium sulfide (Na_2_S·9H_2_O), sodium chloride (NaCl), sodium sulfate (Na_2_SO_4_), allyl isothiocyanate (AITC, purity > 98%) and myrosinase (*β*-thioglucosidase, T4528-25 UN) were purchased from Sigma-Aldrich, Singapore. Phosphate buffered saline (PBS) was purchased from Vivantis Technologies Sdn. Bhd. (Selangor Darul Ehsan, Malaysia).

### 3.2. Extraction and Myrosinase Hydrolysis of GSs

Glucosinolates from different *Moringa* tissues were extracted by following a reported method with minor modifications [38]. Each of the freeze-dried *Moringa* tissues (100 mg) were extracted with 70% methanol (1 mL), samples were heated in a thermomixer at 70 °C for 10 min at 1000 rpm/min, then centrifuged at 4 °C with 10,000× *g* (centrifuge, Sigma 3–18 K, Osterode am Harz, NS, Germany) for 5 min. The supernatant was collected and filtered with 0.45 μm Sartorius filter membrane (Göttingen, Germany). This procedure was repeated three times and the supernatants were combined. The combined supernatants were dried using vacuum centrifugal evaporator (Labconco, Kansas City, MO, USA) and stored at −20 °C before use.

The extracted GSs were suspended in 1 mL of 0.1 M phosphate buffer (pH 6.5) to do the myrosinase (*β*-thioglucosidase) hydrolysis reaction. Ten μL myrosinase solution (24 mg in 100 μL deionized water) was added into each sample. The hydrolysis reaction was conducted in a thermomixer at 25 °C for 4 h with a speed of 500 rpm. The amounts of GSs could be calculated based on the ITCs content, which was measured by means of a calibration curve using sulforaphane as a standard (0–280 μM), as described below.

### 3.3. Extraction of Total ITCs from Different Moringa Tissues

The total ITCs were extracted by following a reported method with minor modifications [41]. Briefly, each freeze-dried *Moringa* tissue (3 g) was firstly extracted with 30 mL hexane and the mixture was shaken for 1 h. This procedure was repeated three times, and the solid residues were then transferred and dried in a fume hood overnight to obtain the defatted samples. After that, 20 mL of ethyl acetate and 10 mL of potassium phosphate buffer (0.1 M, pH 6.5) were added simultaneously to each of the defatted powder and shaken for 2 h. Subsequently, sodium chloride (3.0 g) and anhydrous sodium sulfate (6.0 g) were added to the solution and shaken. The ethyl acetate layer was transferred, and the residual solid was extracted twice with an equal volume of EA; the resulting extracts were combined and dried in a rotary evaporator. The total ITCs extracts were dissolved at 10 mg/mL in DMF and stored in a refrigerator at −20 °C. The sample was further diluted in an aqueous solution; the solution was prepared freshly before use.

Quantification of total ITCs was performed using the cyclocondensation method in a Synergy HT microplate reader from Bio-Tek Instruments Inc. (Winooski, VT, USA). Briefly, 99 μL of a 100 mM K_2_HPO_4_ solution (pH 8.0), 99 μL of methanol, 11 μL of the sample (dilution on demand) and 11 μL of benzene-1,2-dithiol (80 mM, prepared in methanol) or methanol (sample blank) were added in a 0.5 mL micro centrifuge tube and vortex mixed. Samples were incubated at 60 °C for 90 min and allowed to cool to room temperature. Absorbance from four replicates was measured at 365 nm. Quantification was measured by means of a calibration curve using sulforaphane (Sigma-Aldrich) as a standard (0–280 μM). Total ITCs content was expressed as micromoles sulforaphane equivalents per gram of dry weight tissue sample (μmol SE/g DW).

### 3.4. HPLC Method for Separation of GSs and ITCs

Freeze-dried extracted GSs (2 mg/mL) and ITCs (2 mg/mL) were dissolved in water and ethyl acetate, respectively, and filtered through 0.22 μm Sartorius membrane filters prior to injection. Fifteen μL of sample were injected into a Phenomenex C-18 HPLC analytical column (250 mm × 4.6 mm × 4.6 mm i.d. 3 μm) with a guard column made of the same stationary materials and eluted with mobile phase A (0.1% TFA in deionized-water) and phase B (0.1% TFA in methanol). The column temperature was 25 °C and the flow rate was 1.0 mL/min. A gradient elution was performed starting as follows: 0–10 min: 100–80% A, 10–25 min: 80–50% A, 25–40 min: 50–0% A. The column was equilibrated with 100% A for 10 min prior to the next run. Detector wavelength was set at 227 nm (GSs) and 280 nm (ITCs), respectively.

### 3.5. Identification of GSs and ITCs Using LC-MS^2^

Liquid chromatography-mass spectra (LC-MS) were acquired using a Bruker Amazon ion trap mass spectrometer (Middlesex, MA, USA) equipped with a Dionex ultimate 3000 quaternary rapid separation HPLC system (Bannockburn, Lake, IL, USA). The heated capillary and spray voltage were maintained at 250 °C and 8.0 kV, respectively. Nitrogen was operated at 80 psi for sheath gas flow rate and 20 psi for auxiliary gas flow rate. The full scan mass spectra from *m*/*z* 70–1500 were acquired in both positive and negative ion mode with a scan speed at one scan per second. The MS^2^ collision gas was helium with collision energy of 30% of the 5 V end-cap maximum tickling voltage.

### 3.6. Identification of Volatile Compounds from Different Moringa Tissues

The volatile compounds in different *Moringa* tissues were measured using a Shimadzu ultra gas chromatography-mass spectrometer (GCMS-QP2010) coupled with an AOC-5000 Autosampler (Shimadzu Corporation, Kyoto, Japan). A BPX-5 column (30 m × 0.25 mm, 0.25 μm film thickness Scientific Instrument Services, Inc., Ringoes, NJ, USA) was used and the GC temperature programed as follows: the column initial temperature was held at 60 °C for 2 min, followed by an increase to 220 °C at 5 °C/min, and then increased to 280 at 15 °C/min, after that held at 280 min for 10 min. Helium was the carrier gas with the flow of 1.0 mL/min. The injection temperature is 250 °C and injection volume is 1.0 μL in a split mode with a 1:10 ratio. The quadrupoles MS conditions were used as follows: the interface temperature was set at 280 °C and the ionization was produced with 70 eV electron impact at 280 °C. The acquisition mode was full scan (2.78 scan/s) from 6.5 min to 50 min with the mass range *m*/*z*, 50–500. Compounds were identified by comparing their mass spectra with NIST 08 and Wiley 275 MS library and verified by the linear retention index (LRI) values relative to C_10_-C_40_ n-alkane series.

### 3.7. H_2_S Releasing Capacity of Total ITCs from Moringa Samples Using Lead (II) Acetate Paper

The lead (II) acetate paper was prepared by dipping commercial filter paper into 20 mM lead acetate aqueous solution for 30 s, and the pre-treated filter paper was dried at 40 °C under reduced pressure for 24 h [11]. The dried filter paper was cut into 12 mm small circles before use. The cut lead papers were attached on top of the inner cover of each plate.

l-Cysteine solution (10 mM) was prepared by dissolving L-cysteine (36.35 mg) in double strength PBS (27 mL, pH 7.4). The L-cysteine solution (0.9 mL) and ITCs dissolving in DMF (0.1 mL) were gently mixed in each well of the 24-well plate. The plates were incubated at 37 °C for 24 h. During incubation, the produced H_2_S evaporated into the headspace of the microplate and reacted with the lead (II) acetate on the test paper, resulting in the black PbS. The paper was removed from the cover, and its colour was measured with a colorimeter with an 8 mm probe. The results were expressed in Hunter L* (0 = Blank to 100 diffuse white), a* (negative values indicate green, while positive values indicate red) and b* (negative values indicate blue and positive ones indicate yellow) values. The colour intensity (CI) of a sample was calculated by:

CI = SQRT((100 − L*)^2^ + a*^2^ + b*^2^)

To enable data comparison among different samples, the AITC equivalent (AITC-E) was used in this assay. AITC was selected as a standard due to it is naturally occurring in many species of Brassicaceae [12]. Different concentrations of samples or AITC were loaded into 24-well plates. Regression curve for total ITCs of different *Moringa* tissues and AITC were obtained and AITC-E was defined as:

AITC-E = S_sample_/S_AITC_

where S_sample_ and S_AITC_ are the slopes of the linear fitting line of the dose response curves of *Moringa* ITCs and AITC, respectively. To normalize the H_2_S releasing from each *Moringa* tissue, the AITC-E of 100 g DW *Moringa* tissues was calculated by taking the oil yields and the total ITCs yields into consideration.

### 3.8. Statistical Analysis

The results were expressed as means ± standard deviation (n = 3 or 4). Statistical analysis was performed using SPSS^®^ 17.0 for Windows (SPSS Inc., Chicago, IL, USA). The figures were plotted in GraphPad Prism (GraphPad Software, Inc, La Jolla, CA, USA).

## 4. Conclusions

*Moringa* seeds are much stronger H_2_S donors than the leaves and roots, and may have health promotion potential. The H_2_S releasing properties of ITCs extracted from *Moringa* tissues give insights for understanding their biological and pharmacological properties, and therefore, can help to assess and identify the pharmaceutical and nutraceutical potential of naturally occurring GSs and ITCs. For future work, it will be interesting to investigate the actual mechanism of H_2_S release between the reaction of ITCs and L-cysteine.

## Figures and Tables

**Table 1:** Electrospray ionisation mass spectrometry (ESI-MS) of glucosinolates (GSs) in different *Moringa* tissues.

Peak No.	Retention Time (min)	Compounds	*m*/*z* [M − H]^−^	MS^2^
1	9.5	Rhamno-benzyl-GS	570	424, 328, 291
2	18.1	Ac-GS-isomer-I	612	417, 370, 259
3	18.4	Ac-GS-isomer-II	612	416, 370, 259

**Table 2:** ESI-MS of isothiocyanates (ITCs) in different *Moringa* tissues.

Peak No.	Retention Time (min)	Compound	*m*/*z* [M + Na]^+^	MS^2^
1	33.7	Rhamno-benzyl-ITC	334	263, 228, 169, 129
2	34.5	Ac-ITC isomer I	376	359, 270, 211, 151
3	36.5	Ac-ITC isomer II	376	359, 270, 211

**Table 3:** Volatile compounds identified by GC-MS in different *Moringa* tissues.

No.	Compound Name	RT (min) ^$^	LRI ^&^	Identification	Different *Moringa* Tissues Peak Area (%)				
					Seeds with Shell	Seed Kernels	Leaves	Stem	Root
1	Undecane	7.02	1053	LRI, MS	1.17	1.09	1.16	0.57	0.08
2	Benzoic acid, 2,4-bis[(trimethylsilyl)oxy]-	8.81	--	LRI, MS	0.00	0.00	3.32	0.00	0.00
3	Dodecane	11.15	1200	LRI, MS	1.77	1.04	1.43	0.84	0.10
4	Benzene,1,3-bis (1,1-dimethylethyl)-	12.56	1251	LRI, MS	1.47	1.30	1.44	0.75	0.09
5	4-Ethylundecane	12.77	1259	LRI, MS	0.43	0.31	0.41	0.31	0.02
6	Tridecane	13.05	1269	LRI, MS	1.28	1.06	1.40	0.62	0.07
7	Cyclopentasiloxane, dodeca methyl	13.32	--	MS	0.43	0.49	1.92	0.42	0.06
8	2,3,5,8-Tetramethyldecane	14.34	1317	LRI, MS	0.38	0.30	0.41	0.22	0.02
9	m-Tolyl isothiocyanate	16.38	1394	LRI, MS	0.00	0.00	0.00	0.00	79.48
10	Tetradecane	16.53	1400	LRI, MS	0.93	0.80	1.03	0.81	2.05
11	Cycloheptasiloxane, tetradeca methyl	17.51	--	MS	0.14	0.29	0.35	0.29	0.05
12	Pentadecane	18.54	1480	LRI, MS	1.03	1.00	1.10	0.51	0.13
13	Phenol, 3,5-bis(1,1-dimethylethyl)-	19.62	1524	LRI, MS	2.62	2.28	2.68	4.21	0.79
14	Hexadecane	21.42	1599	LRI, MS	21.91	21.47	19.06	24.86	6.54
15	Heptadecane	23.41	1688	LRI, MS	0.57	0.58	0.75	0.44	0.09
16	Octadecane	25.82	1784	LRI, MS	0.41	0.34	0.48	0.52	0.09
17	Hexadecanal	26.57	1836	LRI, MS	0.46	0.21	7.28	0.99	0.04
18	Octadecanal	27.09	1861	LRI, MS	0.17	0.20	1.33	0.24	0.02
19	3,7,11,15-Tetramethyl-2-hexadecen-1-ol	27.46	1880	LRI, MS	0.33	0.31	1.97	0.13	0.10
20	Nonadecane	27.78	1895	LRI, MS	0.39	0.37	0.49	0.30	0.05
21	Dibutyl phthalate	29.36	--	MS	0.00	0.00	0.00	7.26	2.26
22	Hexadecanoic acid	29.45	1981	LRI, MS	4.04	3.32	13.62	5.34	1.00
23	E-Phytol	32.02	2136	LRI, MS	0.00	0.00	1.31	0.00	0.00
24	(*Z*,*Z*)-9,12-Octadecadienoic acid	32.62	--	MS	0.00	0.00	1.43	0.59	0.16
25	Linolenic acid	32.76	--	MS	1.62	1.49	11.09	1.36	0.32
26	Eicosanoic acid	33.13	2269	LRI, MS	2.25	1.67	4.25	2.06	0.28
27	Hexadecanamide	33.57	2310	LRI, MS	7.39	9.92	3.28	4.67	0.64
28	9-Octadecenamide	37.00	2493	LRI, MS	13.21	18.72	6.21	8.95	1.16
29	Octadecanamide	37.38	2519	LRI, MS	7.72	10.41	3.16	4.85	0.65
30	Octadecanoic acid, phenylmethyl ester	38.02	--	MS	0.41	0.39	0.42	0.40	0.12
31	1-Palmitoyl-1,3-propanediol, trimethylsilyl	38.19	--	MS	0.78	0.64	0.63	0.00	0.00
32	Mono(2-ethylhexyl) phthalate	38.83	--	MS	0.72	0.28	0.47	21.50	2.42
33	Heptacosane	39.09	2677	LRI, MS	1.63	1.33	1.37	1.46	0.39
34	Octacosane	40.02	2798	LRI, MS	0.00	0.00	1.12	0.50	0.04
35	Nonacosane	40.87	2911	LRI, MS	0.67	0.54	0.42	3.09	0.46
36	2-(2-Hexyloxyethoxy)ethanol	41.00	--	MS	23.17	17.36	0.00	0.00	0.00
37	Dotriacontane	44.17	3223	LRI, MS	0.50	0.47	0.67	0.64	0.19

^$^ RT: Retention time (min). ^&^ LRI: Linear retention index, which was determined by running a C_10_-C_40_ n-alkane hydrocarbon standard mixture under the same conditions.

**Table 4:** H_2_S releasing capacity of isothiocyanates (ITCs) extracts in different *Moringa* tissues ^&^.

Ranking	Sample Name	Oil Yield (g/100 g *Moringa* Tissue)	ITCs Extract Yield (g/100 g Defatted *Moringa* Tissue)	ITCs Extract Yield (g/100 g *Moringa* Tissue)	AITC-E of *Moringa* ITC Extract (mmol AITC/g of ITC Extract)	AITC-E of *Moringa* Tissue (mmol AITC/100 g of *Moringa* Tissue)
1	Seed kernel	37.28	10.18	6.56	1.56 ± 0.09	9.94 ± 0.58
2	Seed with shell	28.81	6.88	4.90	1.52 ± 0.03	7.49 ± 0.16
3	Root	2.43	3.66	3.57	1.21 ± 0.05	4.34 ± 0.17
4	Leaves	2.82	5.32	5.17	0.57 ± 0.03	2.96 ± 0.18
5	Stem	1.43	2.65	2.61	0.57 ± 0.04	1.49 ± 0.09

^&^: All yield data and AITC-equivalent data are expressed based on dry weight powder; AITC: Allyl isothiocyanate.
